# Lethality and Developmental Delay in *Drosophila melanogaster* Larvae after Ingestion of Selected *Pseudomonas fluorescens* Strains

**DOI:** 10.1371/journal.pone.0012504

**Published:** 2010-09-13

**Authors:** Marika H. Olcott, Marcella D. Henkels, Kise L. Rosen, Francesca L.Walker, Baruch Sneh, Joyce E. Loper, Barbara J. Taylor

**Affiliations:** 1 Department of Zoology, Oregon State University, Corvallis, Oregon, United States of America; 2 Horticultural Crops Research Laboratory, United States Department of Agriculture-Agricultural Research Station, Corvallis, Oregon, United States of America; 3 Department of Botany and Plant Pathology, Oregon State University, Corvallis, Oregon, United States of America; 4 Department of Molecular Biology and Ecology of Plants, The George S. Wise Faculty of Life Sciences, Tel Aviv University, Tel Aviv, Israel; CNRS - Université Aix-Marseille, France

## Abstract

**Background:**

The fruit fly, *Drosophila melanogaster*, is a well-established model organism for probing the molecular and cellular basis of physiological and immune system responses of adults or late stage larvae to bacterial challenge. However, very little is known about the consequences of bacterial infections that occur in earlier stages of development. We have infected mid-second instar larvae with strains of *Pseudomonas fluorescens* to determine how infection alters the ability of larvae to survive and complete development.

**Methodology/Principal Findings:**

We mimicked natural routes of infection using a non-invasive feeding procedure to study the toxicity of the three sequenced *P. fluorescens* strains (Pf0-1, SBW25, and Pf-5) to *Drosophila melanogaster*. Larvae fed with the three strains of *P. fluorescens* showed distinct differences in developmental trajectory and survival. Treatment with SBW25 caused a subset of insects to die concomitant with a systemic melanization reaction at larval, pupal or adult stages. Larvae fed with Pf-5 died in a dose-dependent manner with adult survivors showing eye and wing morphological defects. In addition, larvae in the Pf-5 treatment groups showed a dose-dependent delay in the onset of metamorphosis relative to control-, Pf0-1-, and SBW25-treated larvae. A functional *gacA* gene is required for the toxic properties of wild-type Pf-5 bacteria.

**Conclusions/Significance:**

These experiments are the first to demonstrate that ingestion of *P. fluorescens* bacteria by *D. melanogaster* larvae causes both lethal and non-lethal phenotypes, including delay in the onset of metamorphosis and morphological defects in surviving adult flies, which can be decoupled.

## Introduction

The fruit fly, *Drosophila melanogaster*, is a well-established model organism for the study of host response to microbial infection and animal development. To protect against infection, *D. melanogaster* employs a suite of cellular and humoral defense mechanisms, making it a good reference organism in which to dissect host responses (reviewed in [1], [2], [3], [4], [5], [6]). For example, *D. melanogaster* employ mechanical barriers (such as the cuticle and tightly connected epithelial cells) to reduce the entry of environmental pathogens. In addition, a rapid defense is mounted through the secretion of a battery of inducible effector molecules (i.e. antimicrobial peptides such as Diptericin and reactive oxygen species), activation of phenoloxidase through a complement-like protease cascade (which leads to the production of reactive compounds and melanin), clotting of the hemolymph, and phagocytosis and encapsulation of foreign objects by blood cells (hemocytes), all of which are tightly regulated mechanisms for neutralizing intruders while maintaining a balanced microbiota.

*D. melanogaster* larvae and adults are exposed to a variety of environmental bacteria in their natural habitats. Among these are species of *Pseudomonas*, a diverse genus of gamma proteobacteria commonly found in soil, water, or in association with plants or animals. Certain strains of *Pseudomonas entomophila* [7] and *Pseudomonas aeruginosa* [8], [9] are known pathogens of *D. melanogaster*, but this pathogenicity is thought to be uncommon for the genus. Of 28 strains representing a spectrum of *Pseudomonas* spp. that were tested by Vodovar *et al.* [7], only *P. entomophila* exhibited pathogenicity against *D. melanogaster*. Nevertheless, genes with predicted functions in insect toxicity are present in genomic sequences of other *Pseudomonas* spp., including *fitD* in *Pseudomonas fluorescens* strains Pf-5 and CHA0 [10], and tc-like toxins in *Pseudomonas syringae* pv. *syringae* strain B728a and *P. fluorescens* Pf0-1 [11]. A recent study demonstrated that *fitD* confers strong insecticidal activity, which was exhibited when Pf-5 or the closely-related strain CHA0 was injected into larvae of two insect species, the tobacco hornfworm *Manduca sexta* and the greater wax moth *Galleria mellonella* [10]. These results highlighted the exciting possibility that strains of *P. fluorescens* have additional previously-unappreciated insecticidal activities.

This study was initiated to determine the oral toxicity of *P. fluorescens* against the model organism *D. melanogaster*, and to characterize the effect of the microbial infection throughout host development. The immune response to high doses of pathogenic and non-pathogenic bacteria has been studied extensively in adult *D. melanogaster* and late stage larvae. Very few experiments have examined the impact of exposure to bacteria at earlier developmental stages when the animals are undergoing rapid growth of both body tissues and rapid divisions of the imaginal disc cells, which will generate the adult body. We developed a new protocol to feed timed cultures of *D. melanogaster* larvae with bacterial strains without handling the larvae, hence reducing the risk of injuries and stress prior to infection. Using this non-invasive protocol, we observed three distinct and strain-specific responses of *D. melanogaster* to infection by three strains of *P. fluorescens* (Pf0-1, SBW25 and Pf-5) with fully sequenced genomes [12], [13]. Oral ingestion of strain Pf0-1 had little effect on larvae. In contrast, strains SBW25 and Pf-5 were toxic to *D. melanogaster*, with SBW25 causing a systemic melanization response and Pf-5 causing a dose-dependent lethality and delayed metamorphosis coupled with morphological defects in adults. Induction of an immune response after oral infection with Pf-5, SBW25 and Pf0-1 was also observed by using a *diptericin-lacZ* reporter. Using the novel larval feeding assay developed herein, we could assess the effect of bacterial infection on larval growth rate, development of the imaginal discs and timing of metamorphosis, thereby establishing the toxicity of *P. fluorescens* SBW25 and Pf-5 to *D. melanogaster* and revealing intriguing new insights into the coupled effects of microbial infection on host defense response and development.

## Results

### Pf-5 and SBW25 reduced *D. melanogaster* survival

**Figure 1:**
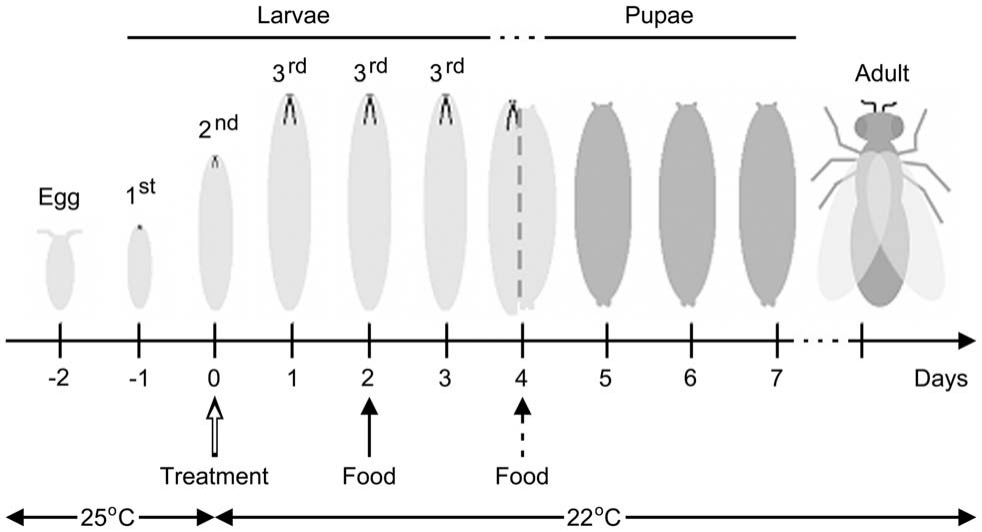
Timeline of the experimental protocol. Above the daily timeline are cartoons of the developmental stage that most closely replicates the control-treatment group trajectory (see Material and Methods for details). On Day −2, eggs were transferred from the egglaying plates to non-nutritive agar plates. The split image on Day 4 indicates the transition period between wandering larvae and prepupal stages around the onset of metamorphosis. Treatments distributed on the agar surface on Day 0 (open arrow) were composed of a yeast suspension, which served as a food source for the larvae, alone (control) or amended with bacterial inoculum. An additional yeast supplement (food) was added on Day 2 to all plates (solid arrow) and every 2 days as long as feeding larvae were visible (dashed arrow). Using this method, larvae were never directly handled and the only disturbance was the application of treatments or food to the surface of the plate. The plates were housed in an incubator on a 12L:12D cycle initially at 25°C; on Day 2, the temperature was shifted to 22°C to slow down larval development.

**Figure 2:**
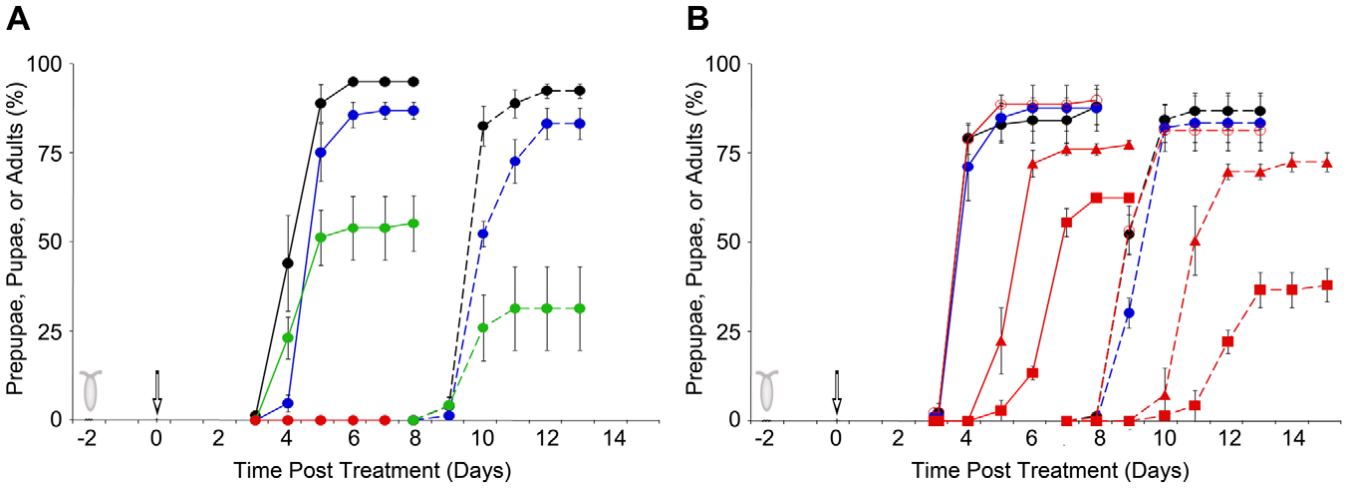
Developmental time course after bacterial inoculation. The percentage of larvae that pupariated, counted as prepupae and/or pupae (solid lines), or emerged as adults (dashed lines) were determined for two different experiments (A and B) in which second instar larvae were fed with a yeast suspension having no bacteria (black) or amended with bacterial strains Pf0-1 (blue), SBW25 (green, Fig. 2A), Pf-5 (red), or a Pf-5 *gacA* mutant (open red circle, Fig. 2B). On the timeline in this and Figure 3, the egg-laying period is denoted by the egg cartoon and the treatment by an open arrow at Day 0. Bacterial treatment as cfu/plate: (A) Pf0-1, 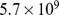; SBW25, 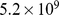; Pf-5, 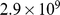, or (B) Pf0-1, 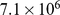; Pf-5 *gacA* mutant, 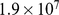; Pf-5 (squares), 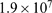; Pf-5 (triangles), 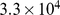.

We determined the effect of three strains of *P. fluorescens* on larval and pupal survival in a newly-developed, non-invasive feeding assay (Fig. 1). Second instar CantonS-A (CS-A) wild-type larvae were fed with a yeast suspension containing high cell densities of bacteria or, as a control treatment, yeast suspension containing no added bacterial cells. In the control treatments, 86±4% of the larvae survived to become adults (Fig. 2, Fig. S1). Similarly, 87±1% of larvae fed with *P. fluorescens* strain Pf0-1 at doses ranging from 10^3^ to 10^9^ cfu/plate survived to adulthood (Fig. 2, Fig. S1), which is not different from the survival of larvae in the control treatment group (Χ^2^ = 0.95, Df = 6, P = 0.99). In contrast, only 30±10% of larvae fed with a yeast suspension containing 10^7^ or 10^9^ cells of SBW25 survived to adulthood (Fig. 2A, Fig. S1), which is significantly different from the control treatment group (Χ^2^ = 61.1, Df = 1, P<0.00001). Insects in the SBW25 treatment died at two different stages: approximately half (58±8%) of the larvae pupariated and, of those pupae, only about half (49±13%) emerged as adults. Thus, ingestion of SBW25 by larvae caused individuals at both feeding and non-feeding stages to die. The consequence of ingesting Pf-5 bacteria was more severe; no larvae fed Pf-5 at the highest dose tested, 10^9^ cfu/plate, pupariated (Fig. 2, Fig. S1).

Other wild-type *D. melanogaster* strains also showed high mortality after feeding with Pf-5 and SBW25 bacteria (Fig. S1; data not shown). When larvae of the Oregon-R (OR) or other two CS wild-type strains were fed Pf-5 at 10^7^ cfu/plate, none pupariated. At a lower dose of 10^4^ cfu/plate, few OR or CS larvae survived to adulthood (3±3% and 7±4%). OR larvae fed SBW25 at either 10^4^ or10^7^ cfu/plate had reduced survival to adulthood (Fig. S1). Thus, ingestion of strains Pf-5 and SBW25 significantly reduced larval and pupal survival rates of *D. melanogaster*.

### Living cells and *GacA* are required for toxicity of strain Pf-5

**Figure 3:**
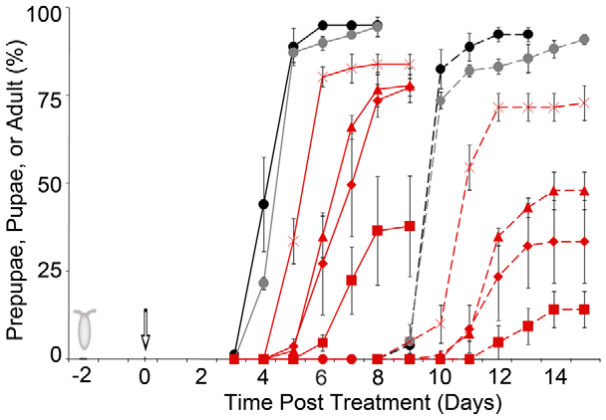
Developmental time course after inoculation with different doses of Pf-5. The percentage of larvae that pupariated, counted as prepupae and/or pupae (solid lines), or emerged as adults (dashed lines) after inoculation with Pf-5 (filled red symbols or star), killed Pf-5 (killed 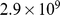 cfu/plate, gray circles) or control (black circles ) treatment were determined for each time point. The Pf-5 cell densities as cfu/plate were 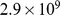 (circles), 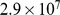 (squares), 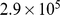 (diamonds), 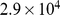 (triangles), 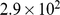 (stars) cfu/plate.

After determining that ingestion of Pf-5 killed larvae, we investigated factors contributing to this toxicity. We first tested whether living Pf-5 cells were required for lethality by feeding larvae heat-killed Pf-5 cells at a dose of 10^9^ cfu/plate. Because 91±1% of larvae survived to adulthood (Fig. 3, Fig. S1), similar to the rate of survival in control and Pf0-1-treatment groups, we conclude that the toxicity of Pf-5 to *D. melanogaster* larvae requires intact live bacterial cells or a heat-labile molecule.

Because the GacS/GacA two component regulatory system controls the production of virulence factors and secondary metabolites in *Pseudomonas* spp. [14] including strain Pf-5 [15], [16], [17], and is required for virulence of *P. entomophila* against *D. melanogaster* [7], we tested the toxicity of a Pf-5 *gacA* mutant. Whereas 90±1% of larvae pupariated, fewer survived to adulthood (81±3%) when fed a yeast suspension containing Pf-5 *gacA* mutant cells at a dose of 10^7^ cfu/plate; only 38±5% of larvae fed the same dose of wild-type Pf-5 survived (Fig. 2B). Thus, GacA had a major effect on the insect toxicity exhibited by Pf-5. The frequency of adult survival of Pf-5 *gacA*-treated larvae (averaging 71±6% for three different experiments at doses of 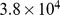, 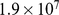, and 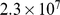
cfu/plate) was found to be significantly lower compared to that of control larvae (Χ^2^ = 7.91, Df = 2, P = 0.02). Thus, we conclude that the lethality associated with ingestion of Pf-5 bacteria by *D. melanogaster* larvae is largely dependent on GacA-regulated functions, but there must also be one or more Pf-5 components unrelated to GacA that also contribute to this lethality.

### Larval and pupal survival after Pf-5-treatment was dose-dependent

Larval survival was strongly dependent on the initial cell density of Pf-5 added to the diet. No larvae survived to later stages when fed with a yeast suspension containing Pf-5 at 10^9^cfu/plate, and both the frequency of pupariation and survival to adulthood increased as the dose of Pf-5 was reduced (Figs 2B, 3, Fig. S1). Chi-squared analysis revealed that the adult survival of larvae fed low doses of Pf-5 (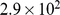 to 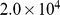
cfu/plate) was not significantly different to that of larvae in the control group (84±3%, Χ^2^ = 2.08, Df = 4, P = 0.71). At intermediate doses of Pf-5 (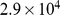 to 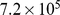 cfu/plate), there was a significant difference in adult survival compared to the control treatment group (62±6%, Χ^2^ = 66.95, Df = 8, P<0.00001). This difference was the result of a significant reduction in the number of pupae surviving to become adults (Figs 2B, 3; Χ^2^ = 37.89, Df = 8, P<0.00001). For the larvae exposed to high treatment doses of Pf-5 (1.7×10^7^ to 2.9×10^9^ cfu/plate), adult survival was significantly reduced (17±4%, Χ^2^ = 229.73, Df = 4, P<0.00001) due to a reduction in both the number of larvae that pupariated and pupae that survived to adult emergence (Χ^2^ = 118.2, Df = 4, P<0.0000; Χ^2^ = 21.1, Df = 4, P<0.00003, respectively). For larvae fed Pf-5 at 10^7^cfu/plate, the frequency of adult survival was highly variable, ranging from 0–38%, suggesting that slight differences in the timing of the bacterial treatment have a larger effect at this high dose than at lower doses. To examine the effect of developmental age, we compared the survival of first and second instar larvae fed the same doses of Pf-5. No first instar larvae fed Pf-5 at 10^4^ or 10^6^ survived to the pupal stage, whereas second instar larvae fed the same doses were able to pupariate (data not shown). Therefore, Pf-5 ingestion caused both larval and pupal mortality in a dose-dependent fashion.

Larvae fed with a yeast suspension containing high doses of Pf-5 tended to die at an earlier stage of development than larvae fed with lower doses. For example, when Pf-5 was added at 10^9^cfu/plate, many larvae died as second instars, some molted into third instar larvae but none were able to pupariate. At doses of 10^4^ to 10^7^ cfu/plate, most larvae survived to become third instars but many were unable to pupariate or died as pre-pupae. Pre-pupae that did not continue development characteristically failed to evert their anterior spiracles or progress beyond the mid-bubble stage and so did not complete pupal ecdysis [18]. At lower doses of Pf-5 (10^2^–10^3^), most larvae pupariated and a few pupae died at later stages of metamorphosis. We found similar survival rates when the total treatment dose was divided into two equal applications on Day 0 and 1 (data not shown) or was delivered as a single dose on Day 0, suggesting that it is the population of bacterial cells that is important, not the feeding regimen, for determining survival. Thus, we have found a strong dose-dependent correlation of survival after ingestion of Pf-5.

### Pf-5, but not Pf0-1 or SBW25, causes developmental delay

**Figure 4:**
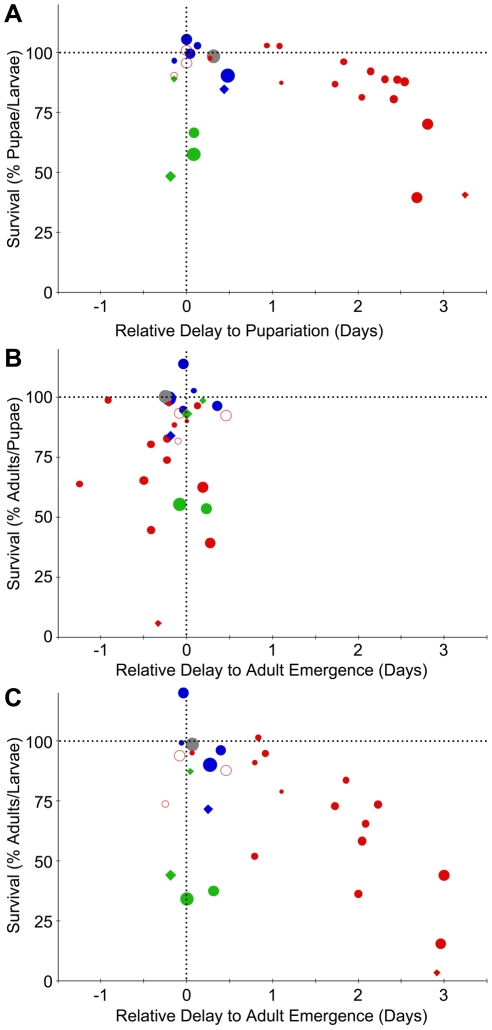
Dose-dependent effects of *P. fluorescens* strains on survival and developmental delay. For each experimental treatment, the percent pupal or adult survival is normalized to that of the appropriate control, which has been set to 100%, and the relative delay to pupariation is normalized to that of the appropriate control, which has been set at 0. In this figure CS-A larval treatment groups are represented using circles and OR larval treatment groups by diamonds. The size of each data point is scaled to the inoculation dose (highest dose/largest circle is 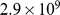 cfu/plate; smallest dose/smallest circle is 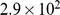 cfu/plate; highest dose/largest diamond is 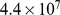; lowest dose/smallest diamond is 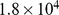); Pf0-1 (blue), SBW25 (green), Pf-5 (red), killed Pf-5 (gray) and a Pf-5 *gacA* mutant (open red circle). (A) The relative developmental delay (see Materials and Methods) during the larval stage (X-axis) was plotted against the percentage of larvae that survived to pupariate (Y-axis) for six different experiments. (B) The relative developmental delay during the pupal stage (X-axis) was plotted against the percentage of pupae that survived to adult survivors (Y-axis). (C) The relative developmental delay during larval and pupal stages (X-axis) was plotted against the percentage of larvae that survived to adult (Y-axis).

We noticed that larvae exposed to Pf-5 treatment frequently failed to pupariate even when viable for many days, suggesting that they were arrested or delayed in their development. In addition, this delay in pupariation also appeared to be dose-dependent. Therefore, we calculated the relative duration of the larval stage for larvae fed different concentrations of Pf-5 (Fig. 4A). Larvae fed Pf-5 at lower doses delayed metamorphosis for up to a day, whereas larvae fed higher doses pupariated with up to a three-day delay. In experiments with high doses of Pf-5 (≥10^7^ cfu/plate), some larvae survived for days but were frequently unable to pupariate. In contrast, the duration of the pupal stage for most Pf-5 treatment groups was similar to that of controls (i.e within a half day), indicating that there is no systematic developmental delay during this stage (Fig. 4B). Thus, as shown in Figure 4C, the overall delay to adult emergence for Pf-5 treatments reflects the delay in pupariation and is accompanied by a reduction in the number of pupae able to eclose as adults. Larvae of other *D. melanogaster* wild-type strains exposed to Pf-5 bacterial treatments also had delayed pupariation. When OR larvae were fed with 4.5×10^4^ cfu/plate Pf-5, pupariation was delayed for three days (Fig. 4A). Larvae from two other CS strains also showed a delay in pupariation (data not shown). The extended, dose-dependent developmental delay is specific to the Pf-5 treatment; larvae fed high doses of SBW25, killed Pf-5, or the Pf-5 *gacA* mutant cells pupariated and emerged as adults at a similar time as control larvae (Fig. 4). Only those CS-A larvae fed the highest dose of Pf0-1 (10^9^ cfu/plate) exhibited any delay in pupariation, and that delay was very slight, whereas larvae fed with lower doses of Pf0-1 pupariated at the same time as control treated larvae (Fig. 4A).

In *D. melanogaster*, the onset of metamorphosis is gated by the attainment of a critical weight in the third instar larval stage in response to hormonal signaling associated with the growth of the imaginal discs, which generate the adult external structures [19], [20], [21], [22]. We measured the surface area of the wing blade as a convenient indicator of body size to determine whether larval developmental delay was correlated with changes in body size. Wing surface area was inversely related to the dose of Pf-5 in the diet (Fig. S2). At 10^7^ cfu/plate, the treatment with the highest cell density of Pf-5 compatible with survival to adulthood, the wing surface area of surviving adults differed significantly from those of control, Pf0-1, and SBW25 treatments, suggesting that the delay in pupariation may be a compensatory mechanism: after exposure to lower populations of Pf-5 cells, the delay allows larvae the time needed to reach their critical weight and normal body size.

### Pf-5 and SBW25 cause structural and behavioral defects in adult flies

Larval ingestion of Pf-5 and SBW25 produced several larval and adult phenotypes in addition to developmental delay and/or lethality. Immediately after the control-,Pf0-1- or SBW25-infested yeast suspensions were added to the assay plates, the larvae remained in place or moved to the region of the plate where the yeast was applied. In contrast, larvae responded to the Pf-5 inoculum by moving away from the site of application towards the edges of the plate. For example, about half of the larvae (61±18%) moved to the side of the dish or wall within two hours of applying a yeast suspension having 10^9^ cfu of Pf-5 to the middle of the plate (data not shown). Although we do not know the nature of the repulsive agent in Pf-5, it was associated with high concentrations of live cells of Pf-5. Larvae did not move away from yeast suspensions having a high dose (10^9^ cfu/plate) of killed Pf-5 cells or a lower dose (≤10^7^ cfu/plate) of viable Pf-5 cells.

Because *D. melanogaster* larvae were found to cease feeding after exposure to *P. entomophila* [7], [23], we tested if they responded the same way to strains Pf-5, SBW25, or Pf0-1. However, when larvae were fed a bacterial suspension containing food dye, the dye was found in the guts of some or most live larvae at one hour and for up to one day, suggesting that larvae continued to feed after exposure to these three *P. fluorescens* strains at the concentrations that we tested (data not shown).

**Figure 5:**
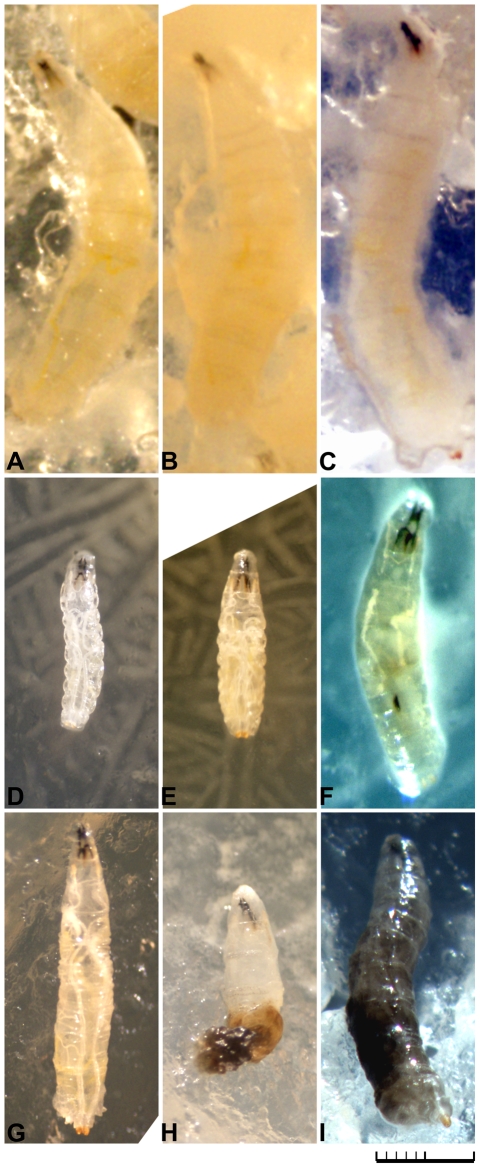
Effects of *P. fluorescens* strains on the morphology of third instar larvae. Third instar larvae on the surface of the treatment plates were photographed using a digital camera mounted on a dissecting scope. Bar is 1 mm with 0.1 mm divisions. (A) 96 hour post control treatment. (B) 72 hour post Pf0-1 treatment (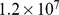 cfu/plate). (C) 72 hour post Pf-5 *gacA* mutant treatment (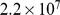 cfu/plate). (D) 48 hour post Pf-5 treatment (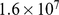 cfu/plate), showing small body size and convoluted longitudinal tracheal trunks. (E) 72 hour post Pf-5 treatment (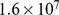 cfu/plate), showing small body size and convoluted tracheal trunks. (F) 72 hour post Pf-5 treatment (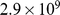 cfu/plate), showing loss of fat body opacity and a melanotic nodule. (G) 72 hour post SBW25 treatment (2.9×10^7^ cfu/plate), with no melanization within the body cavity. (H) 72 hour post SBW25 treatment (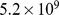 cfu/plate) showing melanization within the posterior third of the body. (I) 72 hour post SBW25 treatment (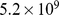 cfu/plate) showing complete melanization reaction.

Larvae fed moderate to high concentrations of Pf-5 (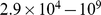
cfu/plate) had a very characteristic appearance that differed from larvae fed control, Pf-5 *gacA* mutant or killed Pf-5 treatments (Fig. 5A–F). After moderate to high Pf-5 treatments, second and third instar larvae were lethargic and the lateral sheets of fat body organ lost their opaque appearance. At death, the larvae were nearly clear and lacked any visible fat body (Fig. 5F). These third instar larvae remained very small and the main tracheal trunks, which stretch dorsally along the full length of the body, had a convoluted trajectory due to the failure of the larvae to extend to full size (Fig. 5D–F). Control third instar larvae typically dig into the agar of the plate in their search for food, whereas the larvae ingesting high doses of Pf-5 (10^7^–10^9^) remained on the surface of the plate.

We observed a striking physical difference in immune response between larvae ingesting SBW25 compared to Pf-5. In the SBW25-treated group, larvae had two fates. About half of the larvae appeared similar to control larvae, whereas the subset of larvae that died developed a systemic melanization reaction followed by death (Fig. 5A, G–I). The first evidence of melanization occurred in the middle of the third instar stage with the appearance of black nodules and epidermal lesions. Subsequently, larvae developed widespread blackening within the body cavity in the posterior abdomen near the malphigian tubules (Fig. 5H). Over the course of the next 6–12 hours, melanization spread throughout the entire body cavity and the animals died swollen and fully extended (Fig. 5I). Larvae that did not develop a melanization reaction successfully pupariated, but in the middle of the pupal stage, about half of these pupae developed a systemic melanization reaction and died. We also noted that many adult flies died post-eclosion after a systemic melanization reaction occurred. By contrast, the Pf-5-treated larvae had the occasional small black nodules associated with tissues or black lesions in the epidermis, but did not show the whole body melanization response exhibited by a subset of larvae that ingested SBW25 (Fig. 5F). In order to determine whether the ingestion of *P. fluorescens* strains induced an immune response, we used a line of *D. melanogaster* expressing β-galactosidase under the control of the promoter of an immune challenge inducible gene encoding an antimicrobial peptide, *diptericin-lacZ*. Second instar larvae were fed Pf0-1, Pf-5 and SBW25 and then dissected as feeding or wandering third instar larvae for β-galactosidase activity. We found that third instar larvae exposed to all three bacterial strains or the Pf-5 *gacA* mutant expressed β-galactosidase in their gut and fat body suggesting that their immune system had been activated (Fig. S3).

**Figure 6:**
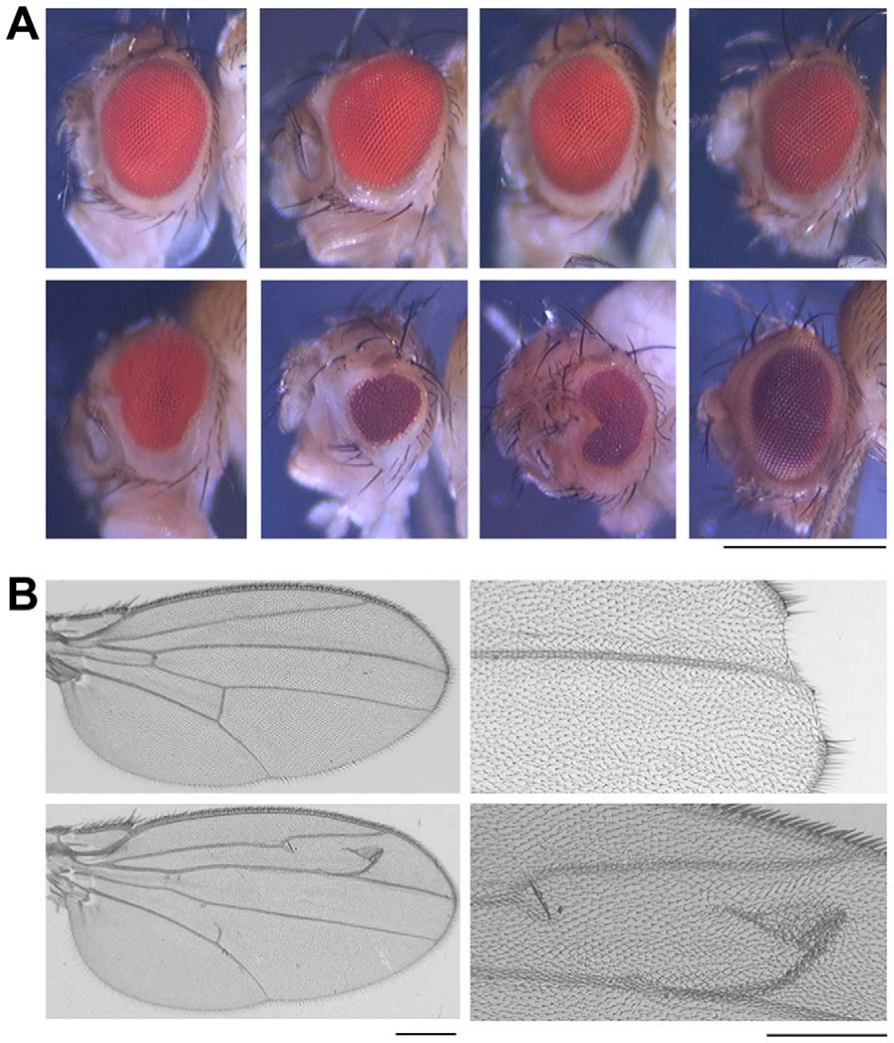
Morphological defects in adult survivors after infection with Pf-5. (A) Eye images from control and bacterial treatment groups. Top panel from left to right: control; 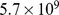 cfu/plate, Pf0-1; 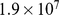
cfu/plate Pf-5 *gacA* mutant; 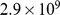 cfu/plate killed Pf-5. Bottom panel from left to right: 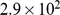 cfu/plate Pf-5; 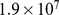 cfu/plate Pf-5; 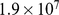
cfu/plate Pf-5; 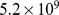 cfu/plate SBW25. Magnification bar = 500 µm. (B) Images of wing blades from adult flies after control (upper left) or Pf-5 at 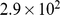 cfu/plate (bottom left, right panel) treatments. Developmental defects visible in these wings include loss of cells at the distal margin (upper right) and/or the presence of ectopic wing veins (bottom left and right). Magnification bar = 100 µm.

The adult survivors of Pf-5 and SBW25 treatments showed morphological defects in specific external structures (Fig. 6, Table S1). In pilot experiments, flattened cuticle preparations of adult survivors of bacterially treated larvae were examined and eyes, head capsule, legs and wings were the only structures in which defects were detected (data not shown). The most common defects in the eye were an abnormally small size and/or the presence of anterior and posterior nicks at the equator, sometimes accompanied by small duplications of bristles and head capsule epidermis (Fig. 6A). A few flies from the Pf0-1-treatment groups had small eye phenotypes but, by Chi-squared analysis, the frequency was not significant compared to the frequency found in the control-treatment group (4%, Χ^2^ = 1.09, Df = 3, P = 0.78). Treatment with any dose of live Pf-5 bacteria caused the majority of the surviving flies to have an eye phenotype that was significantly different from that of the control (91%, Χ^2^ = 245.78, Df = 7, P<0.00001). Similar eye defects were found in survivors of Pf-5 treatment of larvae from the OR or the two other CS strains (Fig. S4). At low to moderate doses of Pf-5, we observed wing defects, such as the presence of extra wing veins and loss of small portions of the wing margin (Fig. 6B), however the average frequency of wing defects spanning all tested doses was not significant (12%, Χ^2^ = 6.50, Df = 7, P = 0.37). On rare occasions, we saw defects in the leg joints but there was no consistent pattern (1%, Χ^2^ = 0.09, Df = 7, P = 0.99). Somewhat surprisingly, unlike the frequency of survival or developmental delay, the frequency of eye and wing defects in Pf-5 treated animals did not show an obvious dose-response relationship. Flies that developed after treatment with Pf-5 *gacA* mutant or heat-killed cells had fewer defects than those treated with live Pf-5 cells (6%, Χ^2^ = 0.77, Df = 2, P = 0.95; 23%, Χ^2^ = 3.40 [n = 1 inoculation dose]; respectively), suggesting that these phenotypes may be a consequence of GacA function. Only the highest dose of SBW25 had a small eye phenotype (87%, Χ^2^ = 17.30, [n = 1 inoculation dose]), suggesting that a differential affect on adult survival and morphology by the three *P. fluorescens* strains accompanies the previously demonstrated distinct survival and developmental delay phenotypes.

### Colonization of *D. melanogaster* larvae by *P. fluorescens*

**Figure 7:**
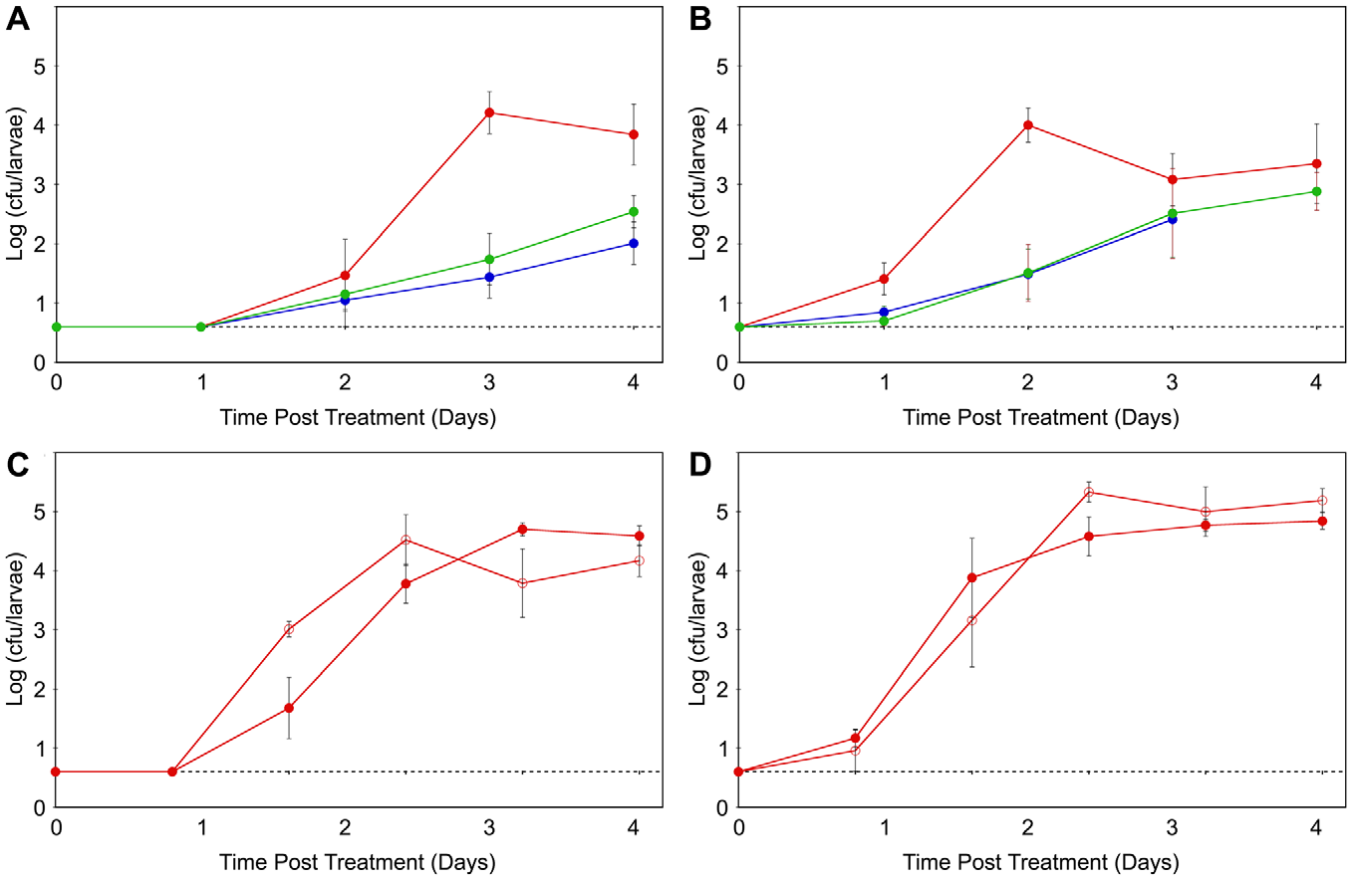
Population sizes of *P. fluorescens* in larvae of *D. melanogaster*. Second instar larvae were inoculated with strains Pf0-1 (blue), SBW25 (green), or Pf-5 (red) (A and B), or the Pf-5 *gacA* mutant (open circle) or wildtype Pf-5 (filled circle) (C and D). Each day following inoculation, larvae were individually surface sterilized, homogenized and bacterial numbers were estimated by culturing. Each point represents the average of five larvae. Bacterial population sizes in the inocula were 10^4^ cfu/plate (A and C), or 10^7^ cfu/plate (B and D). The dotted line denotes the limit of detection. On Day 4, larvae inoculated with Pf0-1 at 10^7^ cfu/plate had pupariated and were not sampled. Error bars denote standard errors.

Because of the dose- and strain-dependent effects of *P. fluorescens* on *D. melanogaster* survival and development, the population sizes of the three strains were assessed from surface-sterilized larvae (Fig. 7). All three strains increased to an internal population size of 10^2^ to 10^5^cfu/larva over the course of the experiment. The population size of Pf-5 bacteria generally exceeded those of SBW25 or Pf0-1. In a second experiment, population sizes of the *gacA* mutant were compared to those of Pf-5 and found to be similar. Therefore, the three strains of *P. fluorescens* colonized internal tissues (including the gut cavity) of the larvae and, at least for strain Pf-5, the capacity to establish internal populations was independent of *gacA*.

## Discussion

In nature, *D. melanogaster* live in an environment full of microorganisms, yet only a few microbes are known to be lethal to wild-type *D. melanogaster* larvae and/or adults, including *Pseudomonas aeruginosa*, *Pseudomonas entomophila*, *Serratia marcescens* and *Beauveria bassiana* ([7], [8], [24], [25]. Here, we demonstrate that the insecticidal activity of *P. fluorescens* strain Pf-5, which was observed previously after injection of the tobacco hornworm *Manduca sexta* and the greater wax moth *Galleria mellonella* [10], is also exhibited when Pf-5 is introduced into *D. melanogaster* through a more natural route including direct contact and feeding of larvae. Strain SBW25 also caused lethality at both feeding and non-feeding stages, but to a lesser extent than Pf-5. As reported previously [26], insect lethality is not universal to *P. fluorescens* strains, and strain Pf0-1 exhibited no detectable toxicity to larvae after ingestion. Nevertheless, ingestion of Pf0-1, like Pf-5 and SBW25, induced an immune response in larvae, assessed using a transgenic line of *D. melanogaster* containing a *diptericin-lacZ* reporter gene fusion. Diptericin plays a critical role in defense of Drosophila against Gram-negative bacteria, including *P. entomophila* [23], being one of the anti-microbial peptides regulated by the Imd immune response pathway. Therefore, as all three strains of *P. fluorescens* were able to induce Diptericin gene expression, the difference in their toxicities may reflect their differential capacities to overcome or thwart different aspects of the immune response of Drosophila. The results of this study place *P. fluorescens* strains Pf-5 and SBW25 in a small group of bacteria that cause mortality of *D. melanogaster* larvae through feeding or physical contact.

Ingestion of either *P. fluorescens* Pf-5 or SBW25 was lethal to larval *D. melanogaster* from two different wild-type strains, but the bacterial strains caused markedly different, dose-dependent host responses. When high doses of Pf-5 were ingested, nearly all larvae died and most failed to molt to the third instar stage. This response is similar to that reported previously for *P. entomophila*: 70% of wild-type third instar larvae died after ingestion of high doses of *P. entomophila* [7]. In the present study, dose-response relationships were also explored, and developmental delay was the most striking consequence of ingestion of lower, sub-lethal doses of Pf-5. Although larvae exposed to sub-lethal doses of Pf-5 pupariated, many of these pupae later died, indicating that the consequences of bacterial ingestion extended into non-feeding stages. In addition, the marked delay in metamorphosis was accompanied by phenotypic changes, such as reduced larval body size and loss of fat body integrity. One of these phenotypes, small larval body size, is also a consequence of ingestion of *P. entomophila* [7]. Occasionally, Pf-5-treated larvae also exhibited a few small melanized nodules, a reaction that is unlikely to be sufficient to cause death. Very rarely, Pf-5-treated larvae turned light brown, but the color change appeared to be post-mortem. In contrast, a striking systemic melanization throughout the hemolymph typically preceded death for the subset of larvae that died following ingestion of strain SBW25. This melanization response is considered to be symptomatic of an overactive immune system, which can be lethal [2]. Correspondingly, in the present study, all larvae or pupae that exhibited systemic melanization later died. Those larvae that did not develop the systemic melanization appeared to develop into pupae at normal rates, indicating that SBW25 did not cause the developmental delay associated with sub-lethal doses of Pf-5.

Many of the developmental and morphological phenotypes associated with Pf-5 ingestion by larvae, including small body size, loss of fat body integrity and prolonged larval development, are reminiscent of larval responses to starvation. During the feeding stages, larvae grow by increasing the size of terminally differentiated polyploid cells in larval tissues, and the adult precursor cells in the imaginal disc undergo numerous divisions. Attainment of normal larval body size has been shown to require continuous acquisition of dietary protein, whereas the continued division of imaginal disc cells is largely independent of nutrition; instead, these cells require circulating growth factors, which are produced by fat bodies [27]. Early in the third instar, larvae assess their critical weight, a checkpoint used to determine the timing at which to initiate metamorphosis [21]. If starved before the critical weight assessment, larvae delay the onset of metamorphosis, whereas larvae starved after this checkpoint metamorphose early and at a smaller size [19], [20]. Wild-type larvae starved from hatching, by feeding only with sucrose, survive for up to a week [28]. Therefore, the three-day delay in metamorphosis and reduced larval body size observed in this study are consistent with a starvation response of larvae at early stages of development prior to the assessment of larval critical weight. Vodovar *et al.* [7] and Liehl *et al.* [23] observed that oral infection with *P. entomophila* caused a cessation in food uptake by third instar larvae of *D. melanogaster*. In our experiments, larvae were provided with sufficient nutrition to complete development and appeared to continue to feed. Therefore, we propose that ingestion of Pf-5 may block larvae from feeding or impair their ability to use or obtain the necessary nutrition to maintain their growth and development. An interesting, although as yet untested, possibility is that Pf-5 ingestion may alter the activity of molecular regulators of larval cell growth and nutrition, such as the insulin receptor/phosphinositol kinase signaling and the TOR (target of rapamycin) pathway, which are known to phenocopy starvation in fed larvae [28], [29], [30]. Alternatively, a delay in morphogenesis can also result from damage to the developing imaginal discs, which regulate critical size in Drosophila [20], [22], and larvae fed the highest doses of Pf-5 compatible with survival produced smaller adult flies as evidenced by atypically small wings. These results suggest that at least some of the delay in metamorphosis may be due to damage or slower cell divisions of the imaginal disc cells in response to Pf-5 infection.

Ingestion of Pf-5 or SBW25 caused specific morphological defects in two different strains of wild-type adult survivors. The defects in the eye, leg and wing discs are similar to those found in adults with mutations in certain genes, such as *cut*, or mutations that disrupt hormonal signaling necessary for initiating pattern formation of the imaginal discs (e.g. [31]). The similarity between the eye and wing phenotypes suggest that ectopic apoptosis of cells within the imaginal discs result in the absence of ommatidia in the eye and loss of wing margin cells in larvae ingesting Pf-5 or SBW25. It is possible that modulation of or interference with the host's immune system by microbial factors could lead to the morphological defects found in Pf-5- and SBW25-treated survivors. For example, peptidoglycan-recognition proteins have been shown to activate the *D. melanogaster* immune system through NF-κB-like factors that can substitute for one another in the developing animal [4], [32]. Furthermore, RNAi experiments of PGRP-SC1/2 in infected *D. melanogaster* larvae led to increased developmental defects, including wing margin defects and lethality [33]. Alternatively, it is possible that bacterial factors interact with other non-immune-related developmental pathways to create the morphological defects we observe.

The three strains of *P. fluorescens* evaluated in this study caused remarkably different host responses when fed to second instar *D. melanogaster* larvae. This observation is not surprising given the enormous genomic diversity of the strains, which share only ca. 60% of their proteomes [13], [34]. Furthermore, strain variation in virulence to *D. melanogaster* is well recognized in *P. aeruginos*a [35], a genus that is far more homogenous than *P. fluorescens* as presently defined [13], [36]. An obvious reason for the differential effects of the *P. fluorescens* strains on the insect is the distinctive genetic compositions of these bacteria. The genomes of Pf-5 and SBW25 each contain ca. 1500–1600 genes that are lineage-specific, having no defined orthologs in the genomes of either the other strain or Pf0-1 [13], [34]. Certain of these lineage-specific genes may have direct roles in the lethality and developmental delay observed in Drosophila following bacteria ingestion. For example, genes for the biosynthesis of at least eight secondary metabolites toxic to certain eukaryotes are among the products of lineage-specific genes in the Pf-5 genome [12], [34]. Among the lineage-specific genes in the SBW25 genome are those encoding a type III secretion system [37], an export pathway found in many Gram-negative bacteria that injects bacterial effectors into the cytosol of eukaryotic cells [38]. The type III secretion apparatus and effectors are key factors in the pathogenicity of *P. aeruginosa* to *D. melanogaster* [39], [40], but this machinery is lacking in *P. fluorescens* strains Pf-5 and Pf0-1 and *P. entomophila* [12], [41]. A key role of Type III secretion is in overcoming host defense response [42], [43], although these pathways represent only one mechanism that bacteria can employ to silence the host immune system, thereby allowing them to invade and colonize the animal. In this study, we found that all three strains of *P. fluorescens*, including the relatively benign strain Pf0-1, became established in the gut cavity or internal tissues of Drosophila larvae and induced host expression of the gene encoding the antimicrobial peptide, Diptericin. Internal population sizes of the lethal strain Pf-5 were higher than those of the other two strains, which could reflect an enhanced capacity to suppress host immune responses, the expanded availability of nutritional substrates as a consequence of host cellular breakdown, or other factors. In *P. aeruginosa*, pathogenicity is mutifactorial, with many bacterial genes contributing to mortality of Drosophila [44]; and combinatorial, with distinct sets of genes required for pathogenicity of different strains [45]. Similar levels of complexity are likely to operate in *P. fluorescens*, and differences in gene regulation as well as composition among the three strains could contribute to their varied effects on host response.

A *gacA* mutant of *P. fluorescens* strain Pf-5 exhibited attenuated toxicity against *D. melanogaster*, but GacA was not required for colonization of internal tissues of the larvae. *gacA* mutants of *P. aeruginosa* [44] and *P. entomophila* [23] also display reduced toxicity against *D. melanogaster*. The GacS/GacA signal transduction system is highly conserved among *Pseudomonas* spp., acting via the positive control of regulatory RNAs to serve as a master regulator of genes encoding for diverse cellular functions, including chronic and acute infection. In *P. fluorescens* Pf-5, the GacS/GacA system controls multiple phenotypes, including secondary metabolite and exoenzyme production, stress response, and motility [15], [16], [17], [46]. Furthermore, a recent transcriptomic analysis identified more than 600 genes expressed under the control of GacA, further highlighting the central role of the GacS/GacA pair in cellular function of the bacterium [16]. Among the genes positively controlled by GacA are those with demonstrated roles in Drosophila lethality of other *Pseudomonas* spp. Examples include *aprA*, which encodes an extracellular metalloprotease that contributes to oral toxicity of *P. entomophila* [23] and *hcnA*, involved in the biosynthesis of hydrogen cyanide, one of many factors contributing to the injectable lethality of *P. aeruginosa* [44], [47]. The GacA regulon of Pf-5 includes many other secondary metabolites, toxins, secretory systems, and exoenzymes that could contribute to the *gacA*-regulated lethality and developmental delay observed herein. Identification of the specific genes and phenotypes functioning in the newly-recognized ingestible toxicity of Pf-5 represents a promising avenue for inquiry that is likely to reveal novel factors functioning in host-microbe interactions.

The most striking finding of this study is the observed link between the physiological response to microbial infection and alterations in the growth, development and differentiation of *D. melanogaster* larvae and adults. A second significant finding is the identification of two strains of *P. fluorescens* that cause mortality of wild-type *D. melanogaster* when ingested. While other groups have focused on the immune response of larvae or adult flies to bacterial infection, we characterized the effects of larval infection on the animals throughout development, thereby identifying novel host responses to bacterial infection. A new non-invasive feeding protocol that mimicked natural routes of microbial infection, which was developed in this study, was key in establishing a link between microbial infection and developmental delay in the *P. fluorescens*-Drosophila interaction. Our study demonstrates strain-specific effects of *P. fluorescens* on *D. melanogaster* morphogenesis and mortality, and opens the door to further investigation into strain-specific and GacA-mediated mechanisms of pathogenicity in a new model system. The combination of complete genomic sequence data, genetic tractability, and sophisticated analytical tools for *D. melanogaster* and *P. fluorescens* will facilitate future characterization of the cellular, molecular and developmental components of the host-microbe interactions observed in this study.

## Materials and Methods

### Bacterial strains and culture conditions

Three strains of *Pseudomonas fluorescens* having fully sequenced genomes were evaluated: strain Pf-5 was isolated from soil in Texas, USA [48]; strain SBW25 was isolated from the phyllosphere of sugar beet in Oxfordshire, England [49]; and strain Pf0-1 was isolated from an agricultural soil in the USA [50]. Cultures of SBW25 and Pf0-1 were obtained from Joseph Raaijmakers, Wageningen University, and Mark Silby, Tufts University. A Pf-5 *gacA* mutant (JL4577) was constructed previously by replacing 626 bp (nt 1 to 626) internal to *gacA* with *aphI*, which confers kanamycin resistance [16]. Inoculum for larval feeding experiments was obtained by culturing strains of *P. fluorescens* on King's Medium B (KMB) [51] and incubating plates overnight at 27°C. Strains were then inoculated into culture tubes containing 5 ml of KMB broth and incubated with shaking (200 rpm) overnight prior to harvest by centrifugation. Cells were washed once, resuspended in sterile deionized water and diluted to an appropriate OD_600_ for each experiment. The initial number of colony forming units (cfu) for each experiment was determined by spreading samples from serial dilutions on KMB.

### *Drosophila melanogaster* cultures

Four isogenic wild-type lines of *D. melanogaster* were used: A CantonS-A (CS-A) line of *D. melanogaster*, obtained from Jeffrey C. Hall, Brandeis University; a Canton-S (CS) line obtained from William W. Mattox, MD Anderson Cancer Center; a Canton-S (CS) line from Jadwiga Giebultowicz, Oregon State University; and an Oregon-R (OR) line from Chris Q. Doe, University of Oregon. All *Drosophila* lines were maintained at 25°C, in a 12 hour light and 12 hour dark cycle (12L∶12D), on standard cornmeal, dextrose, yeast and agar media with Nipagin (*p*-hydroxybenzoic acid methyl ester, Sigma Aldrich, Inc., St. Louis, MO, USA). A fly line containing a *diptericin-lacZ* fusion with isogenic CS-A second and third chromosomes, *y*, *w*, *DDI;+;+* [52] was employed in experiments evaluating the capacity of *P. fluorescens* to trigger an immune response involving activation of the antimicrobial peptide Diptericin by detection of β-galactosidase activity using standard methods.

### Non-invasive insect toxicity assay

To test the toxicity of *P. fluorescens* strains to *D. melanogaster*, we developed a non-invasive assay (Fig. 1), which required transferring insects only at the egg stage, thereby minimizing the risk of stress or wounding prior to infection. To reduce fungal and bacterial contamination, all experiments were performed using autoclaved medium, sterile plates, pipettes, microcentrifuge tubes and ethanol-rinsed utensils. Yeast supplements for larvae and adults were prepared from yeast (Fleischmann's Active Dry yeast, Fenton, MO, USA) microwaved 2–3 times for 30 seconds to kill the yeast cells. To obtain eggs, females were collected as virgins, mated with males for 4–6 days in standard food vials, and then transferred to egg-laying chambers with apple juice agar plates prepared without Nipagin (Apple agar).

The experimental design is outlined in Figure 1. On the first day of the experiment (Day −2), flies were transferred to Petri plates containing Apple agar supplemented by yeast grains (20 mg), and plates were incubated for four hours at 25°C to allow egg lay. From these plates, thirty eggs were transferred aseptically to the surface of non-nutritive agar (2% wt/vol agar in water) having 2–3 mg yeast grains distributed on the agar surface in a 35 mm Petri plate. Plates were incubated at 25°C. The number of first instar larvae per plate was determined on Days −1 and 0 by counting the number of empty egg cases. On Day 0, 200 µl of a yeast suspension was added to the middle of the plate to serve as a food source for second instar larvae. The yeast suspension was prepared by dissolving 0.2 g yeast in 1.2 ml of sterile water or a bacterial suspension, prepared as described above. The plates were transferred to 22°C and, starting at Day 2, larvae were fed with 100 µl of a yeast suspension (0.2 mg yeast/1.2 ml sterile water) at 48 hour intervals as long as live larvae were observed in the dish. Three replica plates were established for every treatment group in the development and survival experiments. The developmental stage was determined at 24-hour intervals and the number of pupae counted until adult emergence. Adult flies were anesthetized with CO_2_ and then fixed in alcohol. Larval development was documented through images captured by a digital camera mounted on a dissecting scope with images adjusted only for contrast and brightness in Photoshop (Adobe Systems Inc., San Jose, CA, USA) as needed.

We established that the assay procedures were sufficient for *D. melanogaster* larval survival and development by assessing the proportion of CS-A eggs that survived in control treatments. In these control plates, 92±0.5% of the eggs transferred to the assay plates (n = 6,928 eggs on 231 plates in seven experiments) hatched into first instar larvae and 86±4% of the larvae survived to adulthood. This level of adult survival is consistent with a null hypothesis expectation of 100% survival to adulthood for larvae in our control treatment regimen by Chi-squared analysis (Χ^2^ = 3.57, Df = 6, P = 0.73359). Based upon this statistical analysis, the procedures for rearing flies were shown to provide an experimental system for assessing the consequence of natural infection of larvae of *D. melanogaster* without introducing artifacts or stress associated with transfer or manipulation of the insect beyond the egg stage of development.

### Calculation of the duration of developmental stages

Because some bacterial treatments lengthened the duration of insect development, we quantified the developmental delay as follows. First, for each experiment, the duration of larval development post-inoculation was empirically determined as the interval (hours) between inoculation and the time when pupariation had reached 50% of the total number of pupae for the control group (T_1/2 pupae control_), based on repeated counts of pupae and prepupae over the course of the experiment (as in Figs 2 and 3). Second, for each treatment group in a given experiment, the duration of larval development was determined as the interval between inoculation and the time when pupariation had reached 50% of the total number of pupae for each treatment (T_1/2 pupae treatment_). For within and between experiment comparisons, the difference between the duration of the larval stage for a bacterial treatment and the duration of the larval stage for control larvae was calculated as 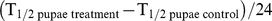 hours/day. Similarly, for each experiment we determined the duration of pupal development as the interval between T_1/2 pupae control_, and the time when 50% of flies had emerged from the pupal case (T_1/2 adult control_). The difference between the duration of pupal development for each treatment group and the duration of the pupal stage for control pupae was calculated as 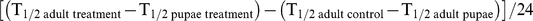 hours/day. The difference in the duration of both larval and pupal stages for each treatment group was calculated as 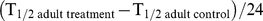 hours/day.

### Bacterial colonization of larvae

Population sizes of the *P. fluorescens* strains internal to larvae of *D. melanogaster* were estimated from surface-sterilized larvae. Six larvae were placed in a drop of the sterilizing solution (2.5 ml bleach and 45 µl 0.01% Triton-X in 10 ml water) for 60 seconds and then transferred serially through four drops of sterile water before being gently blotted and placed individually into a 1.5 ml microcentrifuge tube. Each larva was then homogenized in 50 µl of sterile distilled water, serially diluted, and the dilutions were spread on KMB. Plates were incubated overnight at 27°C prior to counting colonies. The experiment was done twice with similar results, and results from one experiment are presented.

### Morphological defect analysis

All adults that emerged were fixed in ethanol and then examined through a dissecting scope for defects in their external structures. In pilot experiments, flattened adult cuticle preparations were made by macerating the flies in hot 10% KOH solution and placing in a mounting medium (Permount™, Fisher Scientific Research, Pittsburgh PA). These adults were examined for external morphological defects. We discovered morphological defects in eyes, wings and legs. For subsequent experiments, flies that had been fixed in ethanol were examined under a dissecting scope and scored for defects in these structures. Eye defects included smaller eye size and nicks at the edge of the equator. Wing defects included extra or abnormal veins and loss of wing margin cells. Abnormal leg joints were observed in some adults. Digital images of eyes were captured directly into a camera mounted onto a dissecting scope. Final images were only adjusted slightly for contrast and brightness.

Wing size was used as a criterion to estimate adult body size. From each adult fly, one wing was removed and mounted on a slide in glycerol. Images of each wing were captured at 4× and 10× magnification using an Olympus Vanox A2 research grade microscope with a digital camera. Photoshop images were imported into Image Pro 4000 (Media Cybernetics, Bethesda, MD, USA) for surface area measurements. The average wing blade surface area was determined for 10 adult flies from each treatment (except for treatments having low survival rates resulting in less than 10 surviving adults). To evaluate the statistical significance of treatment effects a one-way ANOVA was calculated with the Tukey test for contrast (α = 0.05; P<0.001; F = 7.64) using SigmaStat (Systat Software, Inc.).

## Supporting Information

Table S1 (0.04 MB DOC)

Figure S1 Adult survival after bacterial treatment. Adult survival for each treatment group (circles represent CS-A and diamonds represent OR) is plotted against the treatment dose. For each treatment group, the adult survival is normalized to the survival of the appropriate control, which has been set at 100%. Each point is scaled according to the dose (cfu/plate) for each bacterial treatment. The size of each data point is scaled to the inoculation dose (highest dose/largest circle = 2.9×10^9^ cfu/plate; smallest dose/smallest circle = 2.9×10^2^ cfu/plate; highest dose/largest diamond = 4.4×10^7^; lowest dose/smallest diamond = 1.8×10^4^); Pf0-1 (blue), SBW25 (green), Pf-5 (red), killed Pf-5 (gray) and a *gacA* mutant of Pf-5 (open red circle).
(0.10 MB TIF)

Figure S2 Average wing blade surface area for adult survivors. Lanes (1) control, (2) 5.7×10^9^ cfu/plate Pf0-1, (3) 5.2×10^9^ cfu/plate SBW25, (4) 2.9×10^9^ cfu/plate killed Pf-5, (5) 2.9×10^2^ cfu/plate Pf-5, (6) 2.9×10^4^ cfu/plate Pf-5, 7) 2.9×10^5^ cfu/plate Pf-5, (8) 2.9×10^7^ cfu/plate Pf-5. Asterisk indicates significant difference in wing-surface area from the control. See Materials and Methods for details and calculations.
(0.14 MB TIF)

Figure S3 Diptericin-lacZ expression in third instar larvae from control and bacterial treatments. Fat body cells from third instar *y*,*w DDI* larvae, which were dissected open and then stained for β-galactosidase activity.(A) Segment of larval fat body (delimited by arrows), positioned over body wall musculature, from a control larva in which only a few cells express faint β-galactosidase activity. (B) Labeled fat body cells, positioned over body wall musculature, from a larva fed Pf0-1 at 10^7^ cfu/plate.(C) Isolated segment of larval fat body from a larva fed SBW25 at 10^7^ cfu/plate. (D) Isolated segment of larval fat body from a larva fed Pf-5 at 10^7^ cfu/plate. (E) Isolated segment of larval fat body from a larva fed *gacA* mutant Pf-5 at 10^7^ cfu/plate.
(3.65 MB TIF)

Figure S4 Morphological defects in adult survivors of Pf-5 treated OR and CS adults. Whole mount eye images from OR adult (left panel) inoculated with 4.5×10^4^ cfu/plate Pf-5 and CS adults (middle and right panels) inoculated with 1.7×10^4^ cfu/plate Pf-5. The center of the eye in the right panel was damaged during handling.
(0.80 MB TIF)
