# Supplementary material for: Lethality and Developmental Delay in Drosophila melanogaster Larvae after Ingestion of Selected Pseudomonas fluorescens Strains
Source: PLoS One. 2010 Sep 13;5(9):e12504. doi: 10.1371/journal.pone.0012504 (PMC2938339; doi:10.1371/journal.pone.0012504)
Supplement: Table S1 — (0.04 MB DOC) [file pone.0012504.s001.doc]

**Supplemental data**

**Table SI. Morphological defects in adult flies after infection with various *P. fluorescens* strains in one representative experiment.**

| Treatment | Dose  (cfu/plate) | Number of adults evaluated^a^ | Percent Wing Defects | | | Percent Eye Defects | | | | Percent with defects |
| --- | --- | --- | --- | --- | --- | --- | --- | --- | --- | --- |
|  |  |  | Veins | Cut | Total | Small | Divot | Nick | Total |  |
| Control | NA | 73^c^/74 | 0 | 0 | 0 | 0 | 0 | 0 | 0 | 0 |
| Pf0-1 | 5.7 X 10^9^ | 67^b^/68 | 1 | 0 | 1 | 4 | 0 | 0 | 4 | 6 |
| SBW25 | 5.2 X 10^9^ | 23 | 0 | 0 | 0 | 87 | 0 | 0 | 87 | 87 |
| Pf-5 killed | (2.9 X 10^9^) | 70^b^/71 | 0 | 0 | 0 | 21 | 0 | 6 | 21 | 23 |
| Pf-5 | 2.9 X 10^9^ | 0 | 0 | 0 | 0 | 0 | 0 | 0 | 0 | NA |
| Pf-5 | 2.9 X 10^7^ | 11 | 9 | 9 | 9 | 91 | 27 | 9 | 100 | 100 |
| Pf-5 | 2.9 X 10^5^ | 27 | 11 | 0 | 11 | 96 | 4 | 48 | 96 | 100 |
| Pf-5 | 2.9 X 10^4^ | 31^b^/40 | 23 | 6 | 26 | 100 | 15 | 35 | 100 | 100 |
| Pf-5 | 2.9 X 10^2^ | 59 | 12 | 12 | 24 | 66 | 37 | 44 | 90 | 95 |

^a^ Adult survivors collected after various larval treatments were evaluated for morphological defects. The number of adults evaluated varied depending on the survival rate of larvae to adulthood.

^b^ Number of adults evaluated for morphological wing defects.

^c^ Number of adults evaluated for morphological eye defects.
