# Supplementary figures and images for: Lethality and Developmental Delay in Drosophila melanogaster Larvae after Ingestion of Selected Pseudomonas fluorescens Strains
Source: PLoS One. 2010 Sep 13;5(9):e12504. doi: 10.1371/journal.pone.0012504 (PMC2938339; doi:10.1371/journal.pone.0012504)

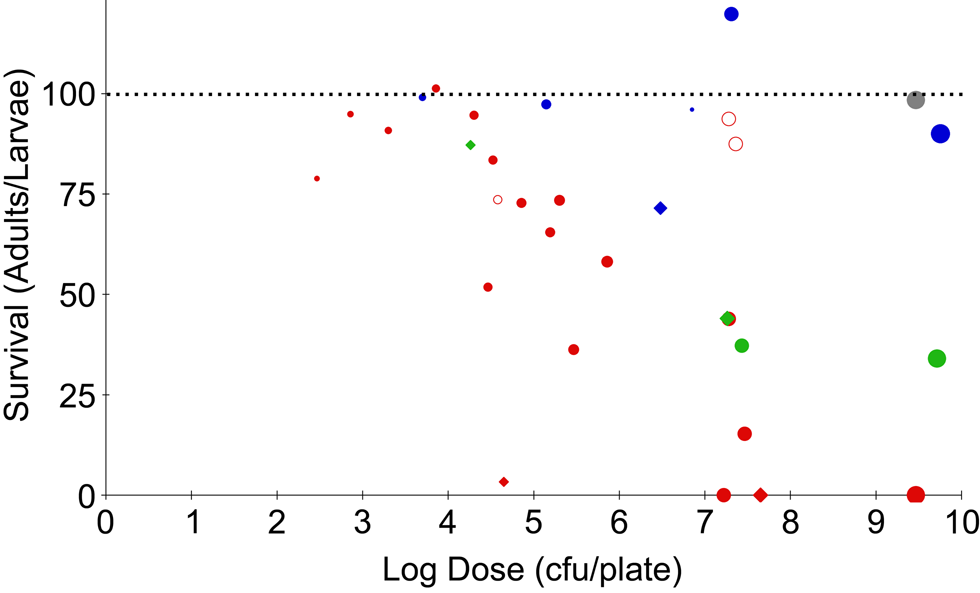

Supplement: Figure S1 — Adult survival after bacterial treatment. Adult survival for each treatment group (circles represent CS-A and diamonds represent OR) is plotted against the treatment dose. For each treatment group, the adult survival is normalized to the survival of the appropriate control, which has been set at 100%. Each point is scaled according to the dose (cfu/plate) for each bacterial treatment. The size of each data point is scaled to the inoculation dose (highest dose/largest circle = 2.9×10^9^ cfu/plate; smallest dose/smallest circle = 2.9×10^2^ cfu/plate; highest dose/largest diamond = 4.4×10^7^; lowest dose/smallest diamond = 1.8×10^4^); Pf0-1 (blue), SBW25 (green), Pf-5 (red), killed Pf-5 (gray) and a gacA mutant of Pf-5 (open red circle). (0.10 MB TIF) [file pone.0012504.s002.tif]

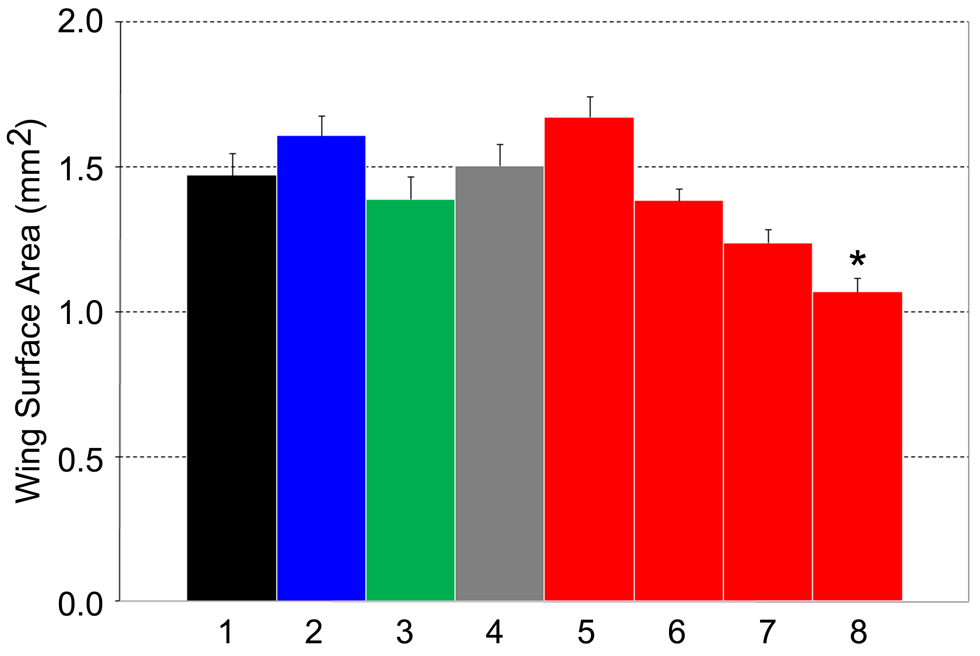

Supplement: Figure S2 — Average wing blade surface area for adult survivors. Lanes (1) control, (2) 5.7×10^9^ cfu/plate Pf0-1, (3) 5.2×10^9^ cfu/plate SBW25, (4) 2.9×10^9^ cfu/plate killed Pf-5, (5) 2.9×10^2^ cfu/plate Pf-5, (6) 2.9×10^4^ cfu/plate Pf-5, 7) 2.9×10^5^ cfu/plate Pf-5, (8) 2.9×10^7^ cfu/plate Pf-5. Asterisk indicates significant difference in wing-surface area from the control. See Materials and Methods for details and calculations. (0.14 MB TIF) [file pone.0012504.s003.tif]

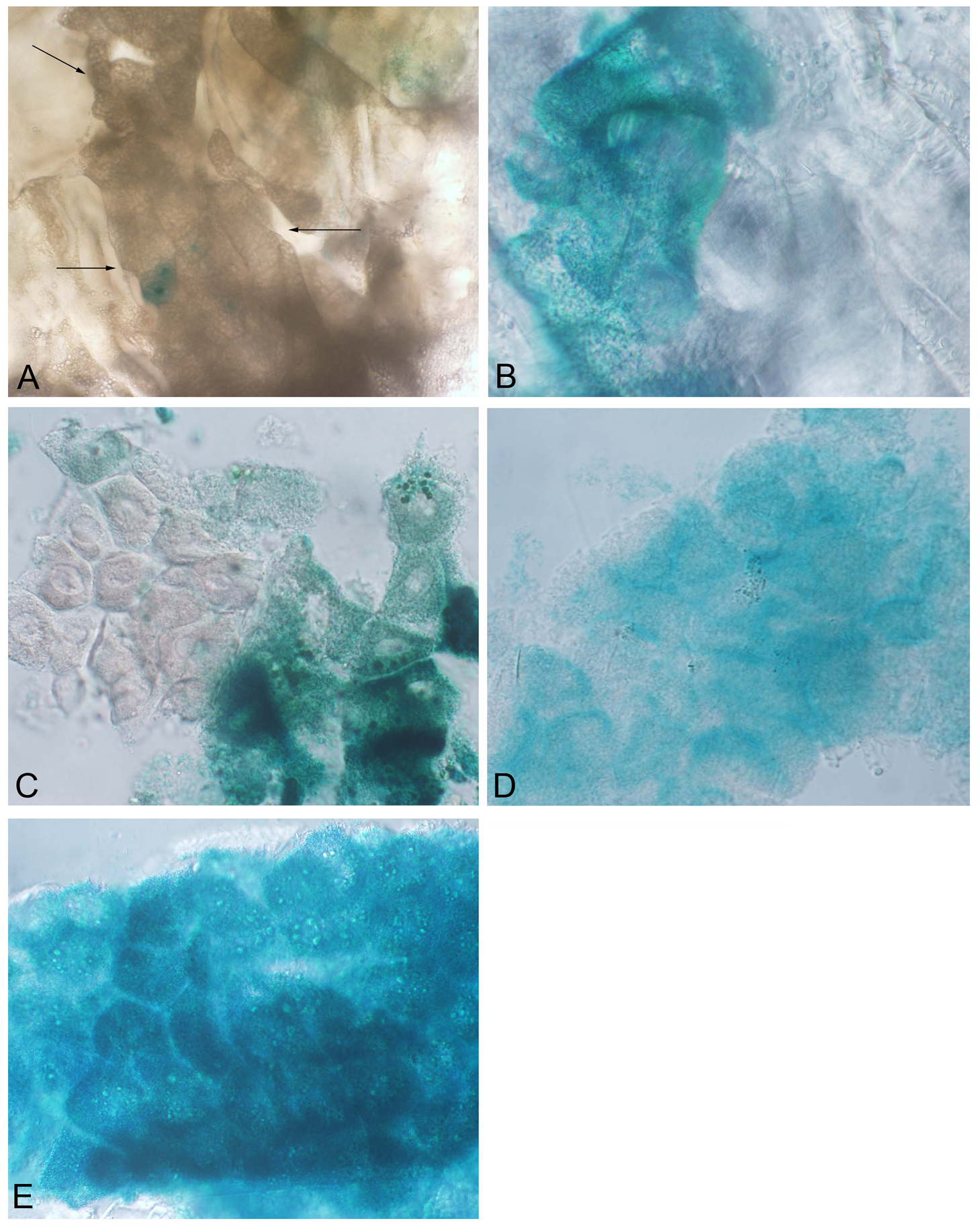

Supplement: Figure S3 — Diptericin-lacZ expression in third instar larvae from control and bacterial treatments. Fat body cells from third instar y,w DDI larvae, which were dissected open and then stained for β-galactosidase activity.(A) Segment of larval fat body (delimited by arrows), positioned over body wall musculature, from a control larva in which only a few cells express faint β-galactosidase activity. (B) Labeled fat body cells, positioned over body wall musculature, from a larva fed Pf0-1 at 107 cfu/plate.(C) Isolated segment of larval fat body from a larva fed SBW25 at 107 cfu/plate. (D) Isolated segment of larval fat body from a larva fed Pf-5 at 107 cfu/plate. (E) Isolated segment of larval fat body from a larva fed gacA mutant Pf-5 at 107 cfu/plate. (3.65 MB TIF) [file pone.0012504.s004.tif]

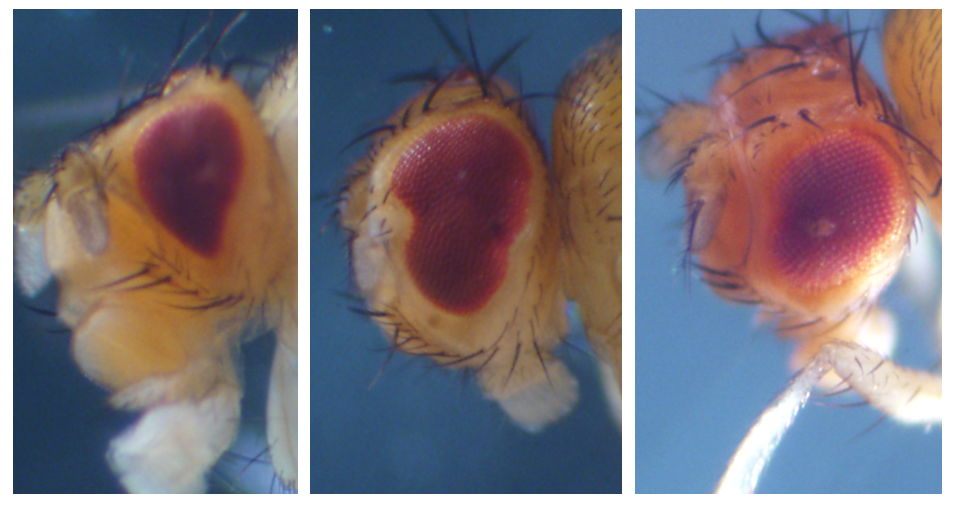

Supplement: Figure S4 — Morphological defects in adult survivors of Pf-5 treated OR and CS adults. Whole mount eye images from OR adult (left panel) inoculated with 4.5×10^4^ cfu/plate Pf-5 and CS adults (middle and right panels) inoculated with 1.7×10^4^ cfu/plate Pf-5. The center of the eye in the right panel was damaged during handling. (0.80 MB TIF) [file pone.0012504.s005.tif]
